# Addressing cervical cancer prevention in Bhutan: A study on the use of loop electrosurgical excision procedures at the primary health care level

**DOI:** 10.1002/puh2.180

**Published:** 2024-05-01

**Authors:** Sangay Tshering, Namkha Dorji, Zimba Letho, Nishal Chhetri

**Affiliations:** ^1^ Department of Obstetrics & Gynecology Jigme Dorji Wangchuck National Referral Hospital Thimphu Bhutan; ^2^ Faculty of Nursing and Public Health Khesar Gyalpo University of Medical Sciences of Bhutan Thimphu Bhutan; ^3^ Department of Pathology and Laboratory Medicine Jigme Dorji Wangchuck National Referral Hospital Thimphu Bhutan

**Keywords:** Bhutan, cervical cancer, cervical intraepithelial neoplasia, human Papillomavirus, loop electrosurgical excision procedure, women's health

## Abstract

**Background:**

Cervical cancer is the top and fourth leading cause of death among women in Bhutan and worldwide, respectively. The cervical cancer elimination flagship program initiated by the Ministry of Health aims to achieve the 90–70–90 goals by 2030. To achieve this, detection and treatment of pre‐cancerous lesions of the cervix through colposcopy and loop electrosurgical excision procedure (LEEP) are being carried out through the outreach health camps.

**Methods:**

This descriptive study aimed to assess the safety and tolerability of LEEP under local anesthesia and assess the margin status of high‐grade lesion (HGL). We analyzed 193 records of clients who underwent LEEP at Wangdue Phodrang Hospital from 26 September to 5 October 2022. The sociodemographic profile, intraoperative pain, complications, and histopathological reports were described using structured pro forma.

**Results:**

The mean age of clients was 40.9 ± 8.5 years (range 25–66 years). There were 3.1% who had intraoperative and 2.6% who had postoperative bleeding. There were 18.6% who had foul‐smelling vaginal discharge, and 8.3% had moderate‐to‐severe intraoperative pain. Histopathological assessment revealed 29.5% with chronic cervicitis, 34.2% with HGL, and 1.6% with microinvasive cervical carcinoma. The margin positivity for HGL was 36.4%.

**Conclusion:**

Given the safety profile of LEEP under local anesthesia in our setting, the scope of providing a complete cervical cancer screening and treatment package at the primary health care level looks promising. Based on the higher rate of overtreatment and margin positivity, we recommend the practical implementation of standard colposcopy guidelines.

## Graphical abstract

LEEP under local anesthesia is safe in our setting. For better accessibility and affordability, LEEP can be extended to the primary care level. The higher rate of overtreatment and margin positivity needs to be addressed at the practice level.

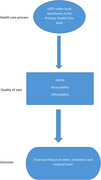

## BACKGROUND

Cervical cancer is the top and fourth leading cause of death among women in Bhutan and worldwide, respectively. An estimated 604,000 cases of cervical cancer were detected, and 342,000 deaths were reported worldwide in 2020 [1]. In Bhutan, cervical cancer presents an age‐standardized incidence of 14.4 and a mortality rate of 8.3 per 100,000 women [2, 3]. Despite the implementation of a national cervical cancer prevention program using conventional Pap smears as early as 2000, data on cervical cancer morbidity and mortality in Bhutan remained static over the last two decades or so [3]. In line with the WHO cervical cancer prevention and elimination strategy, Bhutan is committed to achieving the 90–70–90 goals by 2030, which are 90% human papillomavirus (HPV) vaccination coverage, 70% twice‐lifetime cervical screening, and 90% treatment of preinvasive and invasive cervical lesions. Although primary prevention of cervical cancer with the quadrivalent HPV vaccination in young girls through school vaccination programs and outreach community clinics has been achieved, the focus has shifted to secondary and tertiary prevention through the introduction of HPV screening and timely treatment of precancerous cervical lesions [3].

The Ministry of Health has initiated nationwide mass screening and treatment of the eligible female population between 30 and 65 years old. Although not a screen and treat approach at a single visit, the cervical cancer prevention flagship program has adopted a modified screen and treat approach to maximize the uptake of screening and treatment and minimize loss of follow‐up. Through the outreach health camp setting, all eligible clients were offered high‐risk HPV DNA testing at the nearest primary health center (PHC), and those with positive HPV results were called for colposcopic examination at their respective PHCs.

The clients with colposcopic findings of high‐grade lesion (HGL) as determined by Swede score were either treated on the spot with thermocoagulation (two‐step screen and treat approach) or called for loop electrosurgical excision procedure (LEEP) to their respective district hospitals (three‐step screen and treat approach). The decision to treat with thermocoagulation or LEEP depends on the size of lesion and the type of transformation zone. The Swede score is the colposcopic score allotted by the colposcopist after viewing the cervix with the application of normal saline, 5% acetic acid, and Lugol's iodine, respectively. Acetowhitening, margin status, appearance of atypical blood vessels, lesion size, and iodine staining are accorded a score of 0–2 based on the appearance. A score of ≥5 out of 10 is considered an HGL. A mobile health team composed of gynecologists, scrub nurses, and nurse anesthetists was mobilized to the respective district hospital to conduct the LEEP.

Among the therapeutic modalities used in precancerous cervical intraepithelial neoplasia (CIN), LEEP has been proven to be superior in terms of disease persistence and recurrence [4, 5]. Although cryotherapy and thermocoagulation are emerging treatment modalities used in precancerous lesions of the cervix, we preferred LEEP due to its diagnostic and therapeutic performance. Upon fulfillment of eligibility criteria, the current national protocol mandates the performance of LEEP under total intravenous anesthesia (TIVA). This treatment approach under TIVA requires the client to fast overnight and be admitted to the ward on the morning of the procedure. Post‐procedure care involved 3–4 hours of nil by mouth observation in the ward. The safety and efficacy of LEEP under local anesthesia have been documented in many studies [6, 7]. Postoperative pain, postoperative bleeding, infection, and the margin status of the resected specimen did not differ significantly between the clients who underwent LEEP under local versus general anesthesia [6, 7, 8, 9]. In fact, the local anesthesia group had fewer bleeding complications and higher overall satisfaction [6, 7, 9]. Higher age, higher parity, low level of education, low socioeconomic conditions, widowhood, or separation increased the risk of high‐grade cervical lesion [10, 11].

This study aimed to assess the safety and tolerability of LEEP under local anesthesia and the margin status of HGL in resected LEEP specimens. In doing so, we generate our own local evidence on the safe and effective use of local anesthesia for performing LEEP. Based on the local evidence of safety and effectiveness, the LEEP procedure could be rolled out at the PHC level, thereby benefiting the rural population in terms of accessibility and affordability of health care services.

## METHODS

### Study design and setting

This was a descriptive study with a review of camp records of LEEP performed at Wangdue Phodrang hospital from 26 September to 5 October 2022. The clients were screened using a hybrid capture HPV DNA kit (*digene* HC2 High‐Risk HPV DNA Test, Qiagen). Management was based on the HPV status and colposcopy findings as mentioned in the national guidelines [2]. Clients with pregnancies, ongoing cervicovaginal infections, and menstrual periods were excluded from this study.

The Himalayan kingdom of Bhutan has a population of 0.7 million, with a female population of 346,692 in 2017 [12, 13]. Bhutan has a three‐tiered, free health system, with PHCs at the primary level, district and general hospitals at the secondary level, and three referral hospitals at the tertiary level [12]. Women with gynecologic complaints can access specialist services at the three tertiary hospitals in Thimphu, Monggar, and Gelephu and eight other Emergency Maternal and Obstetric Centers in Wangdue Phodrang, Samtse, Phuentsholing, Tsirang, Samdrupjongkhar, Bumthang, Trashigang, and Lungtenphu Military Hospital. In 2022, Bhutan had 19 obstetrician–gynecologists who were trained in colposcopy.

### Study variables

Data variables were listed and defined according to the study objectives. Complications were categorized into vaginal bleeding and infection. Vaginal bleeding was further divided into intra‐ and postoperative bleeding. Intraoperative bleeding was defined as bleeding that occurred during the procedure and needed hemostatic suturing after failed hemostatic control from ball cauterization and vaginal packing with Monsel solution. Postoperative bleeding was defined as bleeding that occurred after the procedure for 4 weeks and needed hospitalization to achieve hemorrhagic control. Infection was defined as either foul‐smelling vaginal discharge or the presence of fever and lower abdominal pain with or without foul‐smelling vaginal discharge. The verbal pain intensity scale was used to classify pain—no pain, mild, moderate, and severe pain. Margin status of the resected LEEP specimen in CIN was defined as free if the ectocervical, endocervical, or the deep resection margin did not harbor any CIN histopathological findings. If the margins harbored CIN, it was termed positive. If the margin status was not mentioned, it was categorized as inconclusive.

### Data collection

Data on sociodemographic profile (age, gravida, marital status, contraception, education, and occupation, and intraoperative vaginal bleeding complications) were extracted from the camp register into a structured paper‐based pro forma. Data on intraoperative pain, intra‐ and postoperative vaginal bleeding, and infections were extracted from the telephonic follow‐up of clients at second and fourth weeks after the procedure. During the telephonic follow‐up, the identities of the clients were cross‐checked using name, age, and citizenship identity card number. The information was collected by two data collectors using the pro forma. The data on the histopathological reports were collected from the Department of Pathology and entered into the pro forma. Missing data or duplication of data were verified through careful evaluation of the client's name, age, and citizenship identity card and deleted from the final dataset.

### Study procedure

During the registration, the nurse checked and documented the HPV status, colposcopy record, and sociodemographic profiles. The exclusion criteria for LEEP, such as pregnancy, the presence of cervicovaginal infections, and ongoing menstrual period, were ruled out through history‐taking. Upon fulfilling the criteria, the client was provided pre‐procedure counseling about the procedure being performed under local anesthesia, possible pain, bleeding, and vaginal discharge.

**FIGURE 1:**
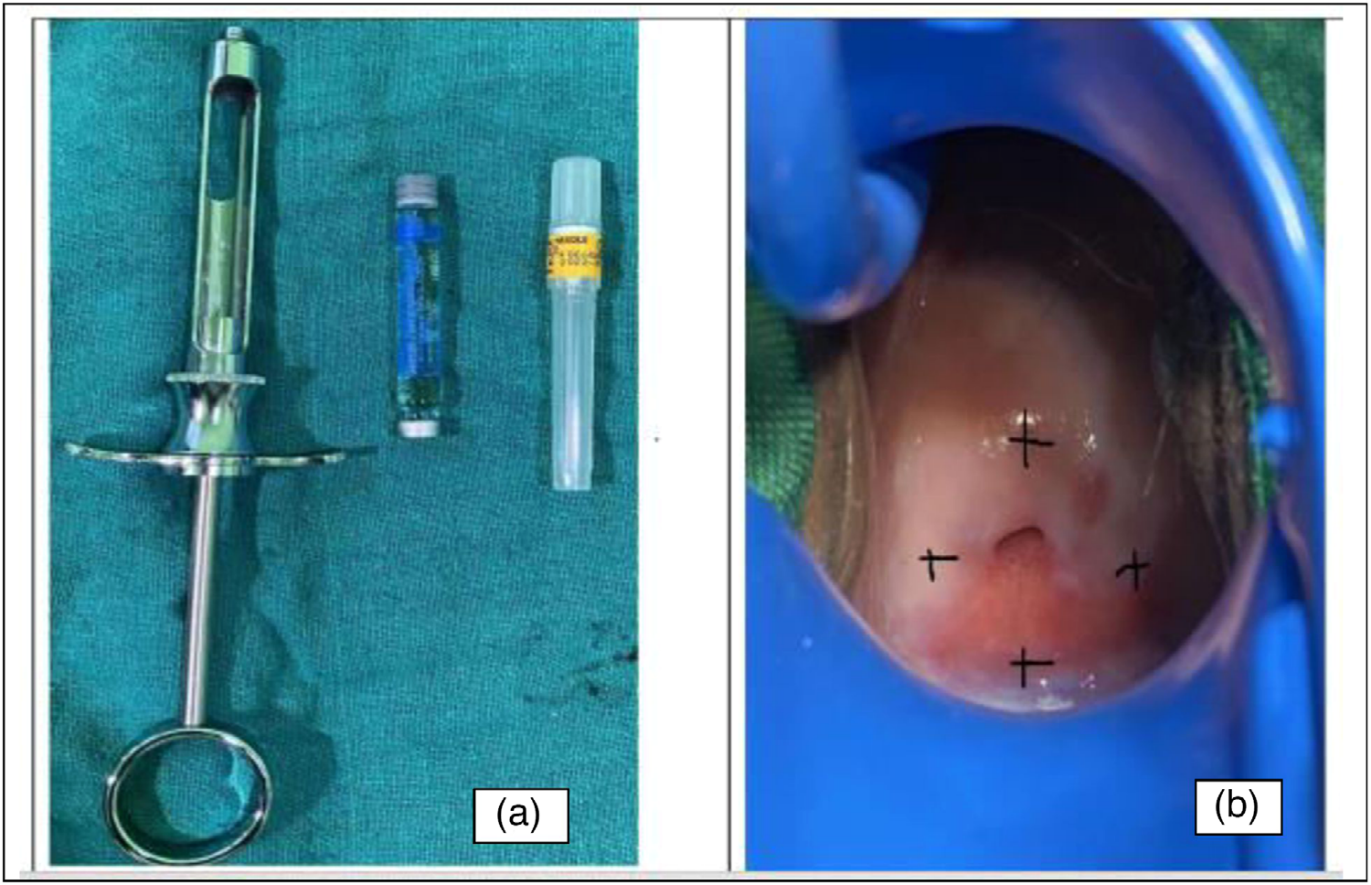
(a) Prefilled dental cartridge and syringe; (b) sites of local anesthesia infiltration on ectocervix marked with black X.

After the written informed consent was signed, the client was placed in lithotomy position, draped, and cervix exposed using an appropriate‐size bivalve speculum with a smoke evacuation system. Using a cotton tip applicator soaked in betadine, the nurse anesthetist cleaned the cervix prior to administering the local anesthesia. A premixed dental cartridge containing lignocaine and adrenaline (Jasocaine‐A DC, Jayson Pharmaceuticals Limited) was infiltrated into the 3, 6, 9, and 12 o'clock positions of the ectocervix using the dental syringe and a disposable needle and waited for 3 min before the excision (Figure 1).

The electrosurgical unit was set at 40–50 W power with blend current, and cervical lesions were cut in one go or in pieces using an appropriate loop size depending on the width of the transformation zone. We relied on the colposcopy diagram to determine the extent of the lesion. Any bleeding from the excision site was coagulated with ball cautery, followed by Monsel paste application and vaginal packing. Bleeding not controlled with these conservative approaches was treated with a hemostatic cervical suture under TIVA.

The client was observed in the post‐procedure room for 30–45 min for any bleeding complications. The clients were also counseled on the minor side effects of LEEP such as watery or minimal bloody vaginal discharge lasting for 4–6 weeks. Warning signs and symptoms on reporting back to the health facility were explained to the client with regard to pelvic infections and heavy vaginal bleeding. Clients were advised to refrain from sexual activity and tub bathing for 4–6 weeks. Tablet paracetamol (1 g) thrice a day for 3 days was prescribed to all clients. All the specimens were labeled with the client's name and citizenship identity card number and transported to the laboratory in 10% phosphate‐buffered formalin.

Reporting of the pathological examination was based on the terminology adopted by the WHO classification of Female Genital Tumors, 5th edition, which describes CIN as HPV‐driven cellular dysplasia of various degrees or viral cytopathic changes with an intact basement membrane and carcinoma as the dysplastic cells invading the basement membrane. CIN grade 1 was defined as the low‐grade lesion and CIN2 and above as HGL.

### Data analysis

Data were double entered, validated, and analyzed using EpiData (version 3.1 for entry and version 2.2.2.183 for analysis, EpiData Association). Additional analysis was performed using STATA (version 13.0, StataCorp LP). The numerical variables were presented as a mean and standard deviation; categorical variables were expressed as frequency and percentage.

### Ethical considerations

Ethics approval was granted by the Research Ethics Board of Health (REBH), Ministry of Health, Bhutan, vide order REBH/Approval/2022/036 dated 21/12/2022. Informed consent to participate was waived off as this study was retrospective in nature. Permission and support for this research were sought from the hospital administrators before initiating the study.

## RESULTS

The mean age of the clients was 40.9 ± 8.5 years (range 25–66 years). The majority (176, 91.2%) of the clients were married; overall, 65.8% (*n* = 127) were third gravida or higher, and 46.6% (*n* = 90) did not use any modern contraceptive methods (Table 1).

**TABLE 1:** Sociodemographic variables of clients who availed loop electrosurgical excision procedure (LEEP) services at Wangdue Phodrang Hospital, Bhutan, September, 2022.

Sociodemographic variables	Frequency (*n*)	Percentage (%)
Age in years		
25–35	60	31.1
36–45	83	43.0
46–66	50	25.9
Gravida		
0	6	3.1
1	20	10.4
2	40	20.7
3	55	28.5
≥4	72	37.3
Marital status		
Unmarried	5	2.6
Married	176	91.2
Divorced	12	6.2
Education		
No education	130	67.4
Nonformal education	5	2.6
School education	43	22.3
Diploma	2	1.0
Graduate and higher	13	6.7
Occupation		
Housewife	151	78.2
Government/Corporate sector	36	18.1
Private sector	6	3.1
Others	1	0.5
Contraception		
None	90	46.6
Contraceptive pills	13	6.7
Intrauterine device	4	2.1
DMPA injection	23	11.9
Tubectomy	41	21.2
Vasectomy	9	4.7
Others	13	6.7

Abbreviation: DMPA, depot medroxyprogesterone acetate.

In terms of bleeding complications, 3.1% (*n* = 6) and 2.6% (*n* = 5) had intra‐ and postoperative bleeding, respectively. In terms of vaginal discharge, 18.6% (*n* = 36) complained of foul‐smelling vaginal discharge; overall, 22.8% (*n* = 44) had brownish vaginal discharge without evidence of infection. In terms of intraoperative pain, 91.7% (*n* = 177) reported no pain to minimal pain after the procedure (Table 2).

**TABLE 2:** Assessment of pain, loop electrosurgical excision procedure (LEEP) complications, and histopathological report in LEEP clients.

Complications	Frequency(*n*)	Percentage (%)
Bleeding complications		
No bleeding	182	94.3
Intraoperative bleeding	06	3.1
Postoperative bleeding	05	2.6
Pain		
No pain	121	62.7
Mild pain	56	29.0
Moderate pain	13	6.7
Severe pain	03	1.6
Vaginal discharge		
Brownish vaginal discharge (without evidence of infection)	44	22.8
Foul smelling vaginal discharge	36	18.6
Bloody/Watery vaginal discharge (without evidence of infection)	113	58.6
Histopathology of resected specimen		
Normal	09	4.7
Chronic cervicitis	57	29.5
CIN 1	58	30.0
High‐grade lesion	66	34.2
Microinvasive carcinoma	03	1.6

Abbreviation: CIN, cervical intraepithelial neoplasia.

Histopathological assessment revealed 29.5% (*n* = 57) chronic cervicitis, 34.2% (*n* = 66) HGL (≥CIN2), and 1.6% (*n* = 3) microinvasive cervical carcinoma (Table 2).

The margin positivity for HGL was 36.4% (*n* = 24) (Table 3).

**TABLE 3:** Assessment of margin positivity in high‐grade lesion (HGL).

Type of HGL	Positive margin (*n*)	Negative margin (*n*)	Inconclusive margin (*n*)	Total (*n*)
CIN 2	3	14	1	18
CIN 3	21	24	3	48
Total (*n*)	24	38	4	66

## DISCUSSION

This study showed that initiating LEEP under local anesthesia is safe in an outreach health camp setting. The overall proportion of intra‐ and postoperative bleeding at 5.7% is similar to the figures reported in other parts of the world (14). In their systematic review, Chamot et al. [14] reported a post‐LEEP bleeding rate of 1.5%–5.2% that required interventions. In our setting, one client underwent hemostatic cervical suturing under general anesthesia, and the rest were managed with the application of Monsel paste and vaginal packing. None of the 11 clients required blood transfusion.

Over 90% of our clients also reported no‐to‐mild pain during the procedure. Only 1.6% of the clients reported severe intraoperative pain, again in concordance with the existing literature rate of 1%–4% [14, 15]. In terms of post‐LEEP infection, our study found that 18.6% of the clients developed localized cervicovaginal infection as clinically manifested by foul‐smelling vaginal discharge. This rate is higher than the previously reported rate of 0%–10% in the literature [14]. Majority of the clients developed some form of vaginal discharge after the LEEP (14). Overall, 22.8% of our clients also reported Monsel paste‐related brownish vaginal discharge without evidence of infection.

In our study, we assumed the higher infection rate was partially due to a misunderstanding of Monsel paste related brownish vaginal discharge as an indicator for infection and also attributable in part to a greater percentage of subclinical chronic cervicitis. None of the clients developed clinical features of systemic pelvic infection requiring hospital admission and intravenous antibiotics. All the clients with localized cervicovaginal infections were treated with oral doxycycline and metronidazole for 7 days as per the syndromic management protocol.

Upon histopathological examination of the LEEP specimen, our study revealed 29.5% of the clients had histopathological evidence of chronic cervicitis. Following LEEP, studies have revealed the proportion of histopathological cervicitis from 2.4% to 37% owing to the differences in the screen and treat approaches adopted and implemented [16, 17]. We expected a higher rate of chronic cervicitis in our study due to the modified screen and treat approach to avoid loss of follow‐up. Similarly, the higher rate of CIN 1 at 29.5% in our study indirectly revealed the modified screen and treat approach leading to overtreatment [18, 19]. Taking the histopathological diagnosis of CIN1 as the upper threshold, our study revealed an overtly high overtreatment rate with LEEP at 65.3%. In the absence of a universally accepted upper threshold rate for overtreatment, it was difficult to compare our overtreatment rate with other studies. However, studies have shown a lower rate of overtreatment at 10% and 11.6%, respectively [18, 20]. Apart from the screen and treat approach, the variation in colposcopic expertise among the colposcopists may have led to this overtly high overtreatment rate in our study [21]. The margin positivity rate of HGL of 36.4% in our study was higher than the 25.25% reported in another study [22]. The reasons for the higher rate of margin positivity in our study could be attributed to the deviations from the standard practice of excisional technique. To minimize margin positivity in the resected specimen, LEEP has to be done with a real time colposcopic view of the type of transformation zone, extent, and depth of the lesion [23]. We relied on the hand‐drawn colposcopic diagram to determine the type of excision, thereby compromising the margin status of the resected specimen.

Bhutan is a signatory to the Alma‐Ata Declaration, which focuses on universal health coverage. Although the health care financing in Bhutan is solely state‐funded, the ever‐increasing health care expenditure is becoming a major hurdle. Moreover, there still exist health inequities and social injustice, especially in the rural population [24]. Owing to geographical terrains, accessibility to health care is difficult and involves substantial hidden costs and out‐of‐pocket expenditure [24]. Although the safety, tolerability, and efficacy of LEEP under local anesthesia have been established from our study, scheduling LEEP at the district hospitals using the three‐step screen and treat approach involved pertinent issues like bed shortages at the hospitals, transportation, and loss of daily wages [24, 25]. Taking LEEP under local anesthesia closer to the rural population would ultimately culminate in positive financial impact at all the levels—client, institution, and the nation as a whole.

The study on LEEP under local anesthesia is the first of its kind in our country. A detailed postoperative complication was studied in this study. Since this was a retrospective study, the true reporting on complications and the subjective pain scale may be distorted due to recall bias. We could not use the more reliable tools to assess intraoperative pain due to the fact that the majority of the clients were illiterate. The efficacy of the intervention in terms of disease persistence or progression in those with positive margin was not assessed, although the margin positivity rate after the LEEP could be a surrogate marker of the residual disease.

## CONCLUSION

Given the safety profile of LEEP under local anesthesia in our setting, the scope of providing a complete cervical cancer screening and treatment package at the primary health care level looks promising. At the policy level, the Department of Obstetrics and Gynecology needs to amend the existing protocol of performing LEEP under TIVA. Based on the higher rate of overtreatment and margin positivity, we recommend the practical implementation of standard colposcopy guidelines.

## AUTHOR CONTRIBUTIONS

*Conceptualization; investigation; writing—original draft; methodology; validation; visualization; writing—review and editing; software; formal analysis; project administration; data curation; supervision; resources*: Sangay Tshering and Namkha Dorji. *Conceptualization; writing—review and editing; software; formal analysis; data curation; validation; methodology; writing—original draft; visualization*: Zimba Letho. *Conceptualization; investigation; writing—original draft; methodology; validation; visualization; writing—review and editing*: Nishal Chhetri.

## CONFLICT OF INTEREST STATEMENT

None of the authors have conflicts of interest to disclose.

## FUNDING INFORMATION

None.

## ETHICS STATEMENT

Ethics approval was granted by the Research Ethics Board of Health (REBH), Ministry of Health, Bhutan, vide order REBH/Approval/2022/036 dated 21/12/2022. Informed consent to participate was waived off as this study was retrospective in nature. Permission and support for this research were sought from the hospital administrators before initiating the study.

## Data Availability

The data that support the findings of this study are available from the corresponding author upon reasonable request.

## References

[1] Sung H , Ferlay J , Siegel RL , et al. Global cancer statistics 2020: gLOBOCAN estimates of incidence and mortality worldwide for 36 cancers in 185 countries. CA Cancer J Clin. 2021;71(3):209‐249. 33538338 10.3322/caac.21660

[2] Reproductive Maternal Newborn Health Program . National Cervical Cancer Guideline. 4th ed. Department of Public Health, Ministry of Health; 2021.

[3] World Health Organization (WHO) . Cervical cancer profile. WHO; 2021. https://www.who.int/publications/m/item/cervical‐cancer‐idn‐country‐profile‐2021

[4] Hurtado‐Roca Y , Becerra‐Chauca N , Malca M . Efficacy and safety of cryotherapy, cold cone or thermocoagulation compared to LEEP as a therapy for cervical intraepithelial neoplasia: systematic review. Rev Saude Publica. 2020;54:27. 32187314 10.11606/s1518-8787.2020054001750 PMC7063859

[5] D'Alessandro P , Arduino B , Borgo M , et al. Loop electrosurgical excision procedure versus cryotherapy in the treatment of cervical intraepithelial neoplasia: a systematic review and meta‐analysis of randomized controlled trials. Gynecol Minim Invasive Ther. 2018;7(4):145‐151. 30306032 10.4103/GMIT.GMIT_56_18 PMC6172872

[6] Lee YJ , Park Y , Lee IO , et al. Delayed hemorrhage effect of local anesthesia with epinephrine in the loop electrosurgical excisional procedure. Obstet Gynecol Sci. 2017;60(1):87‐91. 28217677 10.5468/ogs.2017.60.1.87 PMC5313370

[7] Rezniczek GA , Hecken JM , Rehman S , Dogan A , Tempfer CB , Hilal Z . Syringe or mask? Loop electrosurgical excision procedure under local or general anesthesia: a randomized trial. Am J Obstet Gynecol. 2020;223(6):888. e1‐e9. 10.1016/j.ajog.2020.06.041 32585223

[8] Tzur Y , Berkovitz‐Shperling R , Laskov I , Grisaru D , Michaan N . Recurrent dysplasia after a loop electrosurgical excision procedure: local versus general anesthesia. J Low Genit Tract Dis. 2022;26(4):315‐318. https://journals.lww.com/jlgtd/Fulltext/2022/10000/Recurrent_Dysplasia_After_a_Loop_Electrosurgical.6.aspx 35997172 10.1097/LGT.0000000000000692

[9] Borbolla Foster A , Symonds I . A comparative study of efficacy and outcomes of large loop excision of the transformation zone procedure performed under general anaesthesia versus local anaesthesia. Aust N Z J Obstet Gynaecol. 2012;52(2):128‐132. 22369204 10.1111/j.1479-828X.2012.01420.x

[10] Nessa A , Ara R , Fatema P , et al. Influence of demographic and reproductive factors on cervical pre‐cancer and cancer in Bangladesh. Asian Pacific J Cancer Prev. 2020;21(7):1883‐1889. 10.31557/APJCP.2020.21.7.1883 PMC7573429 32711411

[11] Muwonge R , Ngo Mbus L , Ngoma T , et al. Socio‐demographic and reproductive determinants of cervical neoplasia in seven sub‐Sahara African countries. Cancer Causes Control. 2016;27(12):1437‐1446. 27822586 10.1007/s10552-016-0823-5

[12] Thinley S , Tshering P , Wangmo K , Wangmo K , Wangchuk N . The Kingdom of Bhutan Health System Review. World Health Organization. Regional Office for South‐East Asia; 2017.

[13] National Statistics Bureau . Royal Government of Bhutan. Statistical Yearbook of Bhutan 2021. 39th ed. NSB; 2021. www.nsb.gov.bt

[14] Chamot E , Kristensen S , Stringer JSA , Mwanahamuntu MH . Are treatments for cervical precancerous lesions in less‐developed countries safe enough to promote scaling‐up of cervical screening programs? A systematic review. BMC Womens Health. 2010;10:1‐11. 20359354 10.1186/1472-6874-10-11 PMC2858093

[15] Yap S‐J , Nathan E , Farrell L . LLETZ make it simple: anxiety, pain and treatment outcomes with outpatient large loop excision of the transformation zone under local anaesthesia. Aust New Zeal J Obstet Gynaecol. 2020;60(3):438‐443. https://obgyn.onlinelibrary.wiley.com/doi/abs/10.1111/ajo.13121 10.1111/ajo.13121 32002985

[16] MOK S‐L , YUK JY , HUI S‐K . Outcomes of Patients Undergoing Loop Electrosurgical Excision Procedure for Persistent Low‐grade Abnormal Cervical Smears: A Retrospective Observational Study. Hong Kong J Gynaecol Obstet Midwifery [Internet]. 2014;14(1). Available from: https://hkjgom.org/home/article/view/166

[17] Kasongo N , Kasungu C , Miyoba N , Nyirenda HT , Kumoyo M . Retrospective review of loop electrosurgical excision procedure (LEEP) outcomes at a tertiary hospital in Zambia. Obstet Gynecol Int. 2020;2020:1920218. 32922449 10.1155/2020/1920218 PMC7453247

[18] Kiviharju M , Heinonen A , Jakobsson M , et al. Overtreatment rate after immediate local excision of suspected cervical intraepithelial neoplasia: a prospective cohort study. Gynecol Oncol. 2022;167(2):167‐173. doi:10.1016/j.ygyno.2022.09.016 36153296

[19] Prendiville W , Sankaranarayanan R . Colposcopy and Treatment of Cervical Precancer. IARC Technical Publications; 2017. 33689255

[20] Ebisch RMF , Rovers MM , Bosgraaf RP , et al. Evidence supporting see‐and‐treat management of cervical intraepithelial neoplasia: a systematic review and meta‐analysis. BJOG An Int J Obstet Gynaecol. 2016;123(1):59‐66. 10.1111/1471-0528.13530 26177672

[21] Dorji N , Tshering S , Choden S , et al. Evaluation of the diagnostic performance of colposcopy in the diagnosis of histologic cervical intraepithelial neoplasia 2+ (CIN2+). BMC Cancer. 2022;22(1):1‐8. doi:10.1186/s12885-022-10030-7 36038826 PMC9422165

[22] Feng H , Chen H , Huang D , et al. Relationship between positive margin and residual/recurrence after excision of cervical intraepithelial neoplasia: a systematic review and meta‐analysis. Transl Cancer Res. 2022;11(6):1762‐1769. 35836541 10.21037/tcr-22-1466 PMC9273651

[23] Giannella L , Delli Carpini G , Di Giuseppe J , et al. Should attention be paid to the cone depth in the fully visible transformation zone? Retrospective analysis of 517 patients with cervical intraepithelial neoplasia grade 3. Int J Gynaecol Obstet. 2023;161(1):137‐143. doi:10.1002/ijgo.14520. Epub 2022 Nov 6. PMID: 36263898. 36263898

[24] Tenzin K , Dorji T , Dorji G , Lucero‐Prisno DE III . Health inequities in Bhutan's free healthcare system: a health policy dialogue summary. Public Health Chall. 2022;1:e34. doi:10.1002/puh2.34 PMC12039577 40496697

[25] Tshering S , Dorj N , Monger R , Sonam S , Koirala N . Quality improvement initiative to address bed shortage in the maternity ward at the National Referral Hospital. Health Sci Rep. 2022;5:e721. doi:10.1002/hsr2.721 35821893 PMC9260378

